# The associations between job stress and physical and mental health in firefighters: a systematic review

**DOI:** 10.1186/s12889-026-28923-8

**Published:** 2026-08-11

**Authors:** Fereshteh Kazemi Pakdel, Ahmad Kazemi Pakdel, Morteza Shafi Zadeh, Saeed Abbasi, Omid Tighkar Polkoii

**Affiliations:** 1 Corrective Exercises and Sports Injury Department, Faculty of Physical Education & Sport Sciences, University of Guilan, kilometers10 Rasht-Ghazvin Road, Rasht, 4199613776 Iran; 2 Rasht Branch, Head of Education and Physical Training Department, Rasht Fire Department, Fire Safety Expert and Lecturer, Islamic Azad University, Rasht, Guilan Iran; 3 Hidaj Branch; Operational Commander, Tehran Fire Department, Fire Safety Expert and Lecturer, Islamic Azad University, Tehran, Iran; 4 Master’s Degree in Tehran Fire Department, Fire Safety Expert and Lecturer, Tehran, Iran

**Keywords:** Firefighter, Job Stress, Occupational Stress, Mental Health, Physical Health, Psychosocial Stress

## Abstract

**Background:**

Firefighters are exposed to psychosocial stressors including interpersonal conflict, organizational injustice, and discrimination. This systematic review investigated the associations between work-related psychosocial stress and physical and mental health outcomes among firefighters.

**Methods:**

A comprehensive search was performed in PubMed, PsycINFO, CINAHL, Scopus, Web of Science, and Google Scholar from inception to May 2026. Original cross-sectional, longitudinal, and cohort studies examining associations between job stress and health outcomes in firefighters were included. The review focused on six outcome domains: (1) depression and suicidality, (2) non-depressive mental health problems (PTSD symptoms, anxiety, burnout), (3) alcohol use disorders, (4) sleep quality, (5) somatic disorders (cardiovascular, musculoskeletal, gastrointestinal), and (6) physical performance. Quality assessment was conducted using the Newcastle–Ottawa Scale (NOS). A thematic narrative synthesis was employed due to methodological heterogeneity. Findings were synthesized narratively, with key odds ratios (ORs) extracted and presented in summary tables.

**Results:**

Thirty-four original studies involving 87,242 firefighters across Asia, North America, Europe, and South America were included. The strongest associations were observed for organizational system stress with self-reported depressive symptoms (OR = 8.03 in the longitudinal study), discrimination and harassment with self-reported PTSD symptoms (OR = 2.21) and anxiety (OR = 2.56) in female firefighters, and lack of reward with gastrointestinal disorders (OR = 2.17–2.39) and musculoskeletal disorders (OR = 2.39). Protective factors including social support, self-efficacy, and resilience consistently buffered stress-related health outcomes. Physical performance declined with age (12–13% decrease in strength and power), and less experienced firefighters demonstrated greater physiological stress responses.

**Conclusion:**

Job stress is associated with pervasive and clinically significant adverse health outcomes in firefighters. The available evidence, predominantly cross-sectional, suggests associations rather than causal effects. Organizational interventions targeting workplace fairness, conflict reduction, and anti-discrimination policies, alongside individual-level resilience training, are warranted. Protecting firefighter health through evidence-based stress prevention is an occupational health priority.

**PROSPERO registration number:**

CRD420261417550.

**Supplementary Information:**

The online version contains supplementary material available at https://doi.org/10.1186/s12889-026-28923-8.

## Introduction

Firefighters play an indispensable role in safeguarding public safety, responding to a wide array of emergencies including structural fires, wildland-urban interface fires, hazardous material incidents, medical emergencies, and natural disasters [1]. The nature of firefighting has evolved substantially over recent decades, with modern firefighters assuming responsibilities far beyond traditional fire suppression [2]. These expanded duties, coupled with changes in residential fire dynamics, population growth in wildland-urban interfaces, and shifting demographic patterns, have rendered firefighting an increasingly complex and demanding occupation [3]. Consequently, the occupational environment of firefighters has become the focus of considerable research attention, with investigators documenting elevated risks for a variety of health concerns ranging from thermal injuries and toxicant exposures to cardiovascular events and post-traumatic stress disorder [4].

Much of the early research examining firefighter health focused predominantly on the hazards most intuitively associated with the occupation, including physical, thermal, ergonomic, and chemical risks [5]. More recently, however, an additional aspect of the firefighter work environment has begun to attract growing attention: psychosocial workplace stressors [6]. Job-related psychosocial stress may best be described as mental and emotional strain caused by a combination of workplace events and characteristics, including interpersonal conflicts, organizational fairness concerns, inadequate supervisor support, role ambiguity, work-family conflict, and perceived discrimination or harassment [4]. These psychosocial stressors can be conceptualized along a continuum from acute (e.g., exposure to traumatic critical incidents) to chronic (e.g., persistent organizational injustice or lack of support). Unlike traumatic critical incidents which are acute, time-limited events, chronic psychosocial stressors are often cumulative and embedded within the daily fabric of organizational life [7].

The importance of investigating psychosocial stress in the fire service is underscored by evidence from broader occupational health research demonstrating that these factors can negatively affect health outcomes irrespective of company size, job title, or industry sector [8]. Meta-analytical reviews have shown that exposure to psychosocial stressors prospectively increases the risk of common mental health disorders, stress-related disorders, and a variety of chronic diseases [9, 10]. Furthermore, psychosocial stress has been linked to health-related behaviors associated with chronic disease development, including smoking, alcohol abuse, and physical inactivity [11]. These findings suggest that the associations of psychosocial stress extend beyond psychological distress to encompass physiological dysregulation, somatic morbidity, and maladaptive coping behaviors.

Several characteristics of the firefighting occupation render firefighters particularly vulnerable to psychosocial stress. First, the para-military organizational structure common to many fire services may create rigid hierarchies, limited autonomy, and communication barriers that exacerbate workplace conflicts [12]. Second, the 24-h shift schedules and extended workweeks characteristic of many fire departments disrupt circadian rhythms, reduce opportunities for recovery, and amplify work-family conflict [13]. Third, the culture of stoicism and emotional suppression that permeates many fire services may discourage help-seeking behaviors and reduce the utilization of available mental health resources [14]. Fourth, despite increasing diversification of the fire service workforce, women and racial/ethnic minorities remain underrepresented and may experience heightened levels of discrimination, harassment, and social isolation [15]. These occupation-specific vulnerabilities underscore the need for targeted research examining the associations of psychosocial stress with health outcomes within this unique occupational group.

Despite the growing body of literature examining psychosocial stress in firefighters, the evidence base remains fragmented and methodologically heterogeneous. Investigators have employed diverse measurement tools for assessing both stress exposure and health, complicating cross-study comparisons and meta-analytic synthesis [9, 10]. Furthermore, the predominance of cross-sectional designs limits the ability to draw causal inferences regarding the direction of observed associations [16]. Consequently, there exists a need to systematically identify, appraise, and synthesize the available evidence to provide a comprehensive overview of the health outcomes associated with psychosocial stress in firefighters [17].

Therefore, the objective of this systematic review was to investigate the academic literature to answer the following key research questions: (1) What physical and mental health outcomes are associated with work-related psychosocial stress among firefighters? (2) What is the direction and magnitude of associations between specific psychosocial stressors (e.g., organizational system stress, interpersonal conflict, discrimination, lack of reward) and health outcomes? (3) What protective factors (e.g., social support, self-efficacy, resilience) may buffer the adverse effects of stress? (4) What gaps exist in the current evidence base to inform future research priorities? By synthesizing evidence from original studies across multiple geographic regions, this review provides a comprehensive assessment of the associations between job stress and health outcomes in this essential occupational group.

## Methods

### Study design and registration

This systematic review was conducted in accordance with the Preferred Reporting Items for Systematic Reviews and Meta-Analyses (PRISMA) guidelines to ensure transparency and rigor in reporting [18]. The review was designed as a systematic review with a thematic narrative synthesis approach. The research question was formulated using the PICO framework: Population (active-duty firefighters), Intervention (exposure to work-related psychosocial stress), Comparison (not applicable for this review), and Outcomes (physical and mental health outcomes including depression, PTSD symptoms, anxiety, burnout, alcohol use disorders, sleep quality, somatic disorders, and physical performance). The protocol for this review was registered with PROSPERO (CRD42026141755).

### Eligibility criteria

This systematic review encompassed research assessing the associations of job stress with physical and mental health outcomes in firefighters. The studies that were considered eligible were original research articles published in English from database inception to May 2026. These articles included cross-sectional studies, longitudinal studies, cohort studies, and case–control studies. Studies needed to investigate job-related stress (including occupational stress, psychosocial stress, organizational stress, work-related traumatic events, or perceived discrimination/harassment) as an exposure variable, and assess at least one health-related outcome (physical, mental, or physiological) in firefighter populations. The target population includes active-duty firefighters, with no constraints on age, gender, years of service, or geographic location. Outcomes of interest were categorized into six domains: (1) depression and suicidality, (2) non-depressive mental health problems (PTSD symptoms, anxiety, burnout), (3) alcohol use disorders, (4) sleep quality, (5) physiological parameters and somatic disorders (cardiovascular, musculoskeletal, gastrointestinal), and (6) physical performance (strength, power, endurance, work limitations).

Studies were excluded if they (1) did not measure job stress as an exposure variable, (2) did not include firefighters as the study population (or included firefighters only as a subgroup without separate analysis), (3) focused exclusively on traumatic/critical incident exposure without measuring general occupational or psychosocial stress, (4) were non-original sources (e.g., systematic reviews, narrative reviews, umbrella reviews, conference abstracts, editorials, theses, or book chapters), (5) were published in languages other than English, (6) were qualitative studies without quantitative health outcome measures, or (7) focused exclusively on intervention studies without reporting baseline associations between stress and health outcomes.

For synthesis, studies were classified according to health outcome domain (as listed above). Studies were additionally categorized by study design (cross-sectional versus longitudinal) and geographic region (Asia, North America, Europe, South America) when applicable.

### Sources of information and search strategy

Relevant studies were identified by searching the following electronic databases: MEDLINE (via PubMed), PsycINFO, CINAHL (Cumulative Index to Nursing and Allied Health Literature), Scopus, Web of Science, and Google Scholar. A comprehensive and systematic literature search was conducted from database inception to May 2026. Additionally, the reference lists of pertinent systematic review papers and the included articles were manually screened to identify additional studies that may not have been captured through database searching.

### Search strategy

A comprehensive and systematic literature search was conducted across PubMed/MEDLINE, PsycINFO, CINAHL, Scopus, Web of Science, and Google Scholar databases to identify relevant studies published from database inception to May 2026. The search strategy combined controlled vocabulary terms (e.g., MeSH terms in PubMed) and free-text keywords related to job stress, firefighters, and physical and mental health outcomes. The primary search strategy used in PubMed was as follows:

("firefighter"[Mesh] OR "fire service" OR "fire fighter" OR "fire rescue personnel" OR "first responder") AND ("occupational stress"[Mesh] OR "job stress" OR "work stress" OR "psychosocial stress" OR "work-related stress" OR "organizational stress" OR "job demand" OR "job strain" OR "work pressure" OR "workplace stress" OR "perceived stress" OR "discrimination" OR "harassment" OR "interpersonal conflict" OR "organizational fairness" OR "lack of reward" OR "social support") AND ("mental health"[Mesh] OR "depression"[Mesh] OR "depressive symptoms" OR "suicid" OR "anxiety"[Mesh] OR "post-traumatic stress disorder"[Mesh] OR "PTSD" OR "burnout, professional"[Mesh] OR "burnout" OR "alcoholism"[Mesh] OR "alcohol use disorder" OR "alcohol abuse" OR "sleep"[Mesh] OR "sleep quality" OR "sleep disorder" OR "sleep disturbance" OR "physical health" OR "cardiovascular"[Mesh] OR "blood pressure" OR "heart rate variability" OR "musculoskeletal"[Mesh] OR "back pain" OR "gastrointestinal"[Mesh] OR "gastroesophageal reflux" OR "irritable bowel syndrome" OR "physical performance" OR "physical fitness" OR "work limitation" OR "musculoskeletal disorder*" OR "WMSD").

Equivalent search syntaxes were adapted for the remaining databases according to their indexing systems and search requirements, and Boolean operators (“AND,” “OR”), truncation symbols (*), and database-specific filters were applied where appropriate. The search was restricted to original research articles, human studies, and English-language publications. Additionally, the reference lists of all included articles were manually screened to identify potentially eligible studies not captured through database searching. Two independent reviewers conducted the search process; all identified records were exported into reference management software (EndNote), and duplicate records were removed prior to screening.

### Study selection process

Two reviewers independently screened the titles and abstracts of all retrieved records to identify potentially eligible studies. After the initial screening, the same two reviewers independently assessed the full-text articles against the pre-established eligibility criteria. A third reviewer served as an arbitrator and resolved any disagreements that arose during the screening process. Disagreements were resolved through discussion and consensus, with the third reviewer making the final decision when consensus could not be reached. No automated tools or artificial intelligence-based screening methods were utilized to select potentially eligible studies. The reasons for exclusion of full-text articles were documented and reported in the PRISMA flow diagram.

### Risk of bias assessment (quality assessment)

Quality assessment was conducted according to the methodological design of each included study. Given that the majority of included studies were observational (cross-sectional and longitudinal), methodological quality was assessed using the Newcastle–Ottawa Scale (NOS) adapted for cross-sectional studies [19]. The NOS assessed each study across three major domains: Selection (maximum 5 stars): including representativeness of the sample, sample size justification, description of non-respondents, and ascertainment of the exposure (job stress) using validated measurement tools. Comparability (maximum 2 stars): assessing whether the study controlled for important confounders (e.g., age, gender, years of service, shift schedule, or other relevant covariates) in the design or statistical analysis. Outcome (maximum 3 stars): including assessment of the health outcome using validated measurement tools and the appropriateness of statistical tests. Based on the total NOS score, studies were classified into three quality categories: Good (8–10 stars), Satisfactory (6–7 stars), and Poor (0–5 stars) [19]. Following this classification, Dodge, Coles [20] received 5 stars and was classified as "Poor." Two independent reviewers performed all quality assessments, and disagreements were resolved through discussion and consensus. Studies classified as "Poor" were not excluded from the review but were noted as having high risk of bias, and sensitivity analyses were considered to assess their impact on overall findings.

### Data extraction process

Two reviewers independently extracted data from all included studies using a standardized data extraction form developed in Google Sheets, based on the Cochrane Consumers and Communication Review Group data extraction template [21]. A third reviewer cross-checked the extracted data to ensure accuracy and completeness. No attempts were made to contact study authors for missing data unless the missing information was deemed essential for the primary analysis (e.g., key demographic data or effect sizes). The following information was extracted from each included study: study characteristics (source, author, year, study design, sample size, sex distribution, age, participant characteristics); exposure variables (type of job stress measured, measurement tools used); exposure characteristics (duration of exposure assessment); and outcome measures (health outcomes assessed, specific measurement tools, key findings including odds ratios, correlation coefficients, or other effect sizes where reported).

## Results

### Study selection

**Fig. 1:**
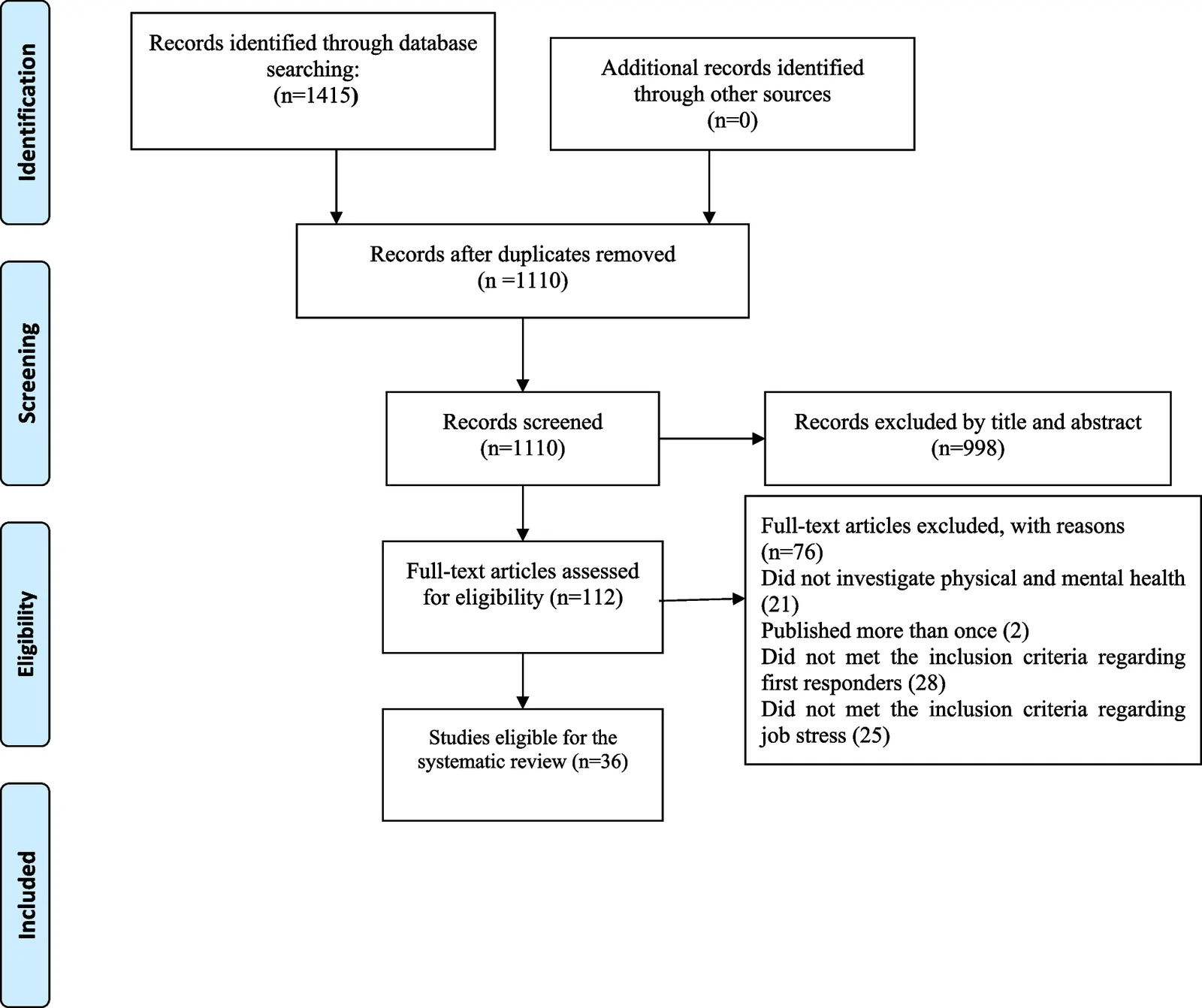
Flow diagram of systematic literature search

The systematic search initially identified 1,415 records from the electronic databases (MEDLINE, PsycINFO, CINAHL, Scopus, Web of Science, and Google Scholar). After removing 305 duplicate records, 1,110 distinct studies were subjected to title and abstract screening. Of these, 998 were removed for irrelevance, primarily because they did not measure job stress as an exposure variable, did not include firefighters as the study population, were review articles or non-original sources, or focused exclusively on traumatic/critical incident exposure without measuring general occupational or psychosocial stress. Following full-text review of the remaining 112 articles, an additional 76 were excluded based on the pre-established eligibility criteria. Finally, following full-text review and assessment against the eligibility criteria, a total of 34 original studies were included in this systematic review (Fig. 1).

### Synthesis of outcomes

A quantitative meta-analysis was not possible because the study designs (predominantly cross-sectional), exposure measurements, health outcome assessments, and reported results (e.g., odds ratios, correlation coefficients, prevalence estimates) were highly heterogeneous across the included studies. Heterogeneity was identified through qualitative assessment during data extraction and quality appraisal, focusing on substantial differences in clinical (e.g., populations, sports contexts, risk factors), methodological (e.g., study designs, validation approaches, sample sizes), and statistical aspects (e.g., diverse measures, reported outcomes). No formal statistical quantification of heterogeneity (e.g., I^2^ statistic) was performed, as the studies were too clinically and methodologically diverse to support meaningful pooling of effect estimates in a meta-analysis. As a result, a thematic narrative synthesis method was used to compile, compare, and analyze the results. This approach initially categorized the studies into six thematic groupings according to the primary health outcome domains: (1) depression and suicidality, (2) non-depressive mental health problems (PTSD symptoms, anxiety, burnout), (3) alcohol use disorders, (4) sleep quality, (5) physiological parameters and somatic disorders, and (6) physical performance. A summary table (Table 3) was created to organize findings by outcome domain, presenting key effect sizes (e.g., odds ratios, correlation coefficients) for rapid comparison across studies. The main results of each study were then compiled, compared, and discussed in writing within their own thematic framework. A detailed summary table (Table 1) presents the key characteristics and primary findings of each included study to allow for rapid comparison across studies.

**Table 1:** General description of the samples included in the individual studies

Source, year	Study design	Sample size	Sex	Age [years (mean ± SD or range)]A	Participants characteristics	Exposure/Intervention	Exposure characteristics	Outcomes measure (specific tools)
**1. Depression & Suicidality**								
An et al. (2015) [22]	Longitudinal (panel)	538	Male	20–29 (8.2%), 30–39 (30.9%), 40–49 (46.5%), 50 + (14.5%)	Male municipal firefighters (South Korea)	Organizational system stress (fairness, support, position appropriateness)	Baseline and follow-up assessment/1 year	Self-reported depressive symptoms (CES-D)
Saijo et al. (2007) [23]	Cross-sectional	1,672 (563 analyzed)	1,479 M, 193 F	37.1 ± 9.6 (total)	Japanese firefighters	Job stress (workload, intergroup conflict)	Cross-sectional/NR	Self-reported depressive symptoms (CES-D ≥ 16)
Saijo et al. (2008) [24]	Cross-sectional	563	Mixed	37.3 ± 9.5	Japanese firefighters (24-h shift workers)	Job stress (workload, intergroup conflict, supervisor support)	Cross-sectional/NR	Self-reported depressive symptoms (CES-D), job dissatisfaction
Payne & Kinman (2019) [25]	Cross-sectional	909	85% M, 15% F	29 and under (9%), 30–39 (28%), 40–49 (40%), 50–59 (21%), 60 + (2%)	UK firefighters (7 Fire and Rescue Services)	Relationship conflicts, job demands, job resources	Cross-sectional/NR	Work-related depression (depression-enthusiasm scale), work-related anxiety (anxiety-contentment scale)
Jahnke et al. (2019) [26]	Cross-sectional	1,646	Female	18–29 (10.5%), 30–39 (29.6%), 40–49 (37.2%), 50–59 (20.8%), 60 + (1.8%)	Female firefighters (US)	Perceived discrimination and harassment	Cross-sectional/NR	Self-reported depressive symptoms (PHQ-8), anxiety, self-reported PTSD symptoms, alcohol abuse
Carpenter et al. (2015) [27]	Cross-sectional	311	94% M, 6% F	38.24 ± 11.40	Professional firefighters (US)	Occupational stress, social support	Cross-sectional/NR	Suicidal ideation (SBQ-R)
Park et al. (2019) [28]	Cross-sectional	31,435	30,595 M, 840 F	M: 42.3 ± 8.8, F: 38.7 ± 7.9	Korean firefighters (nationwide)	Perceived job stress	Cross-sectional/NR	Suicidal ideation (single item)
Stanley et al. (2018) [29]	Cross-sectional	1,027	72.5% M, 27.5% F	38.91 ± 11.10	US firefighters	Occupational stress, distress tolerance	Cross-sectional/NR	Suicidality (SBQ-R)
Zegel et al. (2025) [30]	Cross-sectional	273	92.3% M	40.4 ± 9.7	US firefighters (large metropolitan area)	Resilience, distress tolerance (DT), distress overtolerance (DO)	Cross-sectional/NR	Anxiety (GAD-7), depression (PHQ-9)
Rapisarda et al. (2024) [31]	Longitudinal	274	Mixed	38.44 ± 8.96	Canadian firefighters (Montreal, 67 stations)	PTEs, work-related stressors, social support, coping styles	Longitudinal (2 weeks intervals)/NR	Self-reported depressive symptoms, self-reported PTSD symptoms, generalized anxiety symptoms
**2. Non-depressive mental health problems (PTSD, Anxiety, Burnout)**								
Mitani et al. (2006) [32]	Cross-sectional	1,013	Mixed	20–29 (4.1%), 30–39 (12.5%), 40–49 (23.3%), 50–59 (11.7%)	Japanese fire service workers	Job-related stress, traumatic exposure	Cross-sectional/NR	Self-reported PTSD symptoms (IES-R), burnout (MBI)
Saijo et al. (2012) [33]	Cross-sectional	1,667	1,474 M, 193 F	M: 37.1 ± 9.6, F: 33.6 ± 8.2	Japanese urban firefighters	Job stress (by KOSS)	Cross-sectional/NR	Self-reported PTSD symptoms (IES-R)
Jahnke et al. (2019) [26]	Cross-sectional	1,646	Female	18–29 (10.5%), 30–39 (29.6%), 40–49 (37.2%), 50–59 (20.8%), 60 + (1.8%)	Female firefighters (US)	Perceived discrimination and harassment	Cross-sectional/NR	Self-reported PTSD symptoms (PCL-C), anxiety
Smith et al. (2018) [34]	Cross-sectional	639	93.6% M	38.5 (mean)	US urban career firefighters	PTSD severity, sleep disturbance	Cross-sectional/NR	Alcohol use severity (AUDIT), alcohol use coping reasons
Angelo & Chambel (2015) [35]	Longitudinal (cross-lagged panel)	1,007	74% M, 26% F	37.1 ± 7.8	Portuguese firefighters	Organizational demands	Baseline and 6-month follow-up	Burnout (OLBI)
Smith et al. (2019) [36]	Cross-sectional	639	93.6% M	38.5 (mean)	US firefighters	Work pressure, work stress, work-family conflict	Cross-sectional/NR	Burnout (MBI)
Makara-Studzińska et al. (2019) [37]	Cross-sectional	366	Male	20–30 (14.2%), 31–40 (42.1%), 41–50 (33.1%), 51–60 (10.6%)	Polish male firefighters	Life stress (general)	Cross-sectional/NR	Burnout (LBQ)
**3. Alcohol Use Disorders**								
Kim et al. (2018) [38]	Cross-sectional	7,151	Mixed (6,484 M, 667 F)	M: 42.3 ± 8.8, F: 38.7 ± 7.9	Korean firefighters	Perceived job stress (KOSS)	Cross-sectional/NR	Alcohol use problems (AUDIT-K)
Hosoda et al. (2012) [39]	Cross-sectional	311	Male	20–29 (10.6%), 30–39 (29.6%), 40–49 (36.0%), 50–59 (23.8%)	Japanese male firefighters	Poor perception of workplace environment	Cross-sectional/NR	Alcohol dependence
Jahnke et al. (2019) [26]	Cross-sectional	1,646	Female	18–29 (10.5%), 30–39 (29.6%), 40–49 (37.2%), 50–59 (20.8%), 60 + (1.8%)	Female firefighters (US)	Perceived discrimination and harassment	Cross-sectional/NR	Alcohol abuse
Arbona et al. (2017) [40]	Cross-sectional	232	Male	Black: 38.17, Latino: 37.90	Black and Latino male firefighters (US)	Perceived general stress (PSS)	Cross-sectional/NR	Alcohol abuse
**4. Sleep Quality**								
Haddock et al. (2013) [41]	Cross-sectional	660	95% M	41.9 ± 10.5	US central region firefighters	Shift length (48-h vs 24-h), general life stress	Cross-sectional/NR	Excessive daytime sleepiness (EDS), sleep quality
Lim et al. (2014) [42]	Cross-sectional	31,435	Male	42.3 ± 8.8	Korean male firefighters	Occupational stress factors (by KOSS)	Cross-sectional/NR	Poor sleep quality (PSQI)
Yook (2019) [43]	Cross-sectional	329	Male	30–39 (27.5%), 40–49 (56.7%), 50 + (15.8%)	Korean male firefighters	Occupational stress (KOSS)	Cross-sectional/NR	Sleep quality (PSQI)
**5. Physiological Parameters & Somatic Disorders**								
Choi et al. (2016) [44]	Cross-sectional	560	Mixed	Range 20–65	Professional firefighters (US)	Increased job demands, 24-h work shifts	Cross-sectional/NR	Elevated blood pressure (systolic BP)
Shin et al. (2016) [45]	Cross-sectional	434	Mixed	20–29 (5.3%), 30–39 (35.9%), 40–49 (52.5%), 50 + (6.2%)	Korean firefighters	Occupational climate, organizational system stress	Cross-sectional/NR	Heart rate variability (HRV)
Yook (2019) [43]	Cross-sectional	329	Male	30–39 (27.5%), 40–49 (56.7%), 50 + (15.8%)	Korean male firefighters	Occupational stress (KOSS—8 factors)	Cross-sectional/NR	HRV (LF, HF, LF/HF ratio), cardiorespiratory fitness (Bruce protocol), arterial stiffness (baPWV)
Kim et al. (2016) [46]	Cross-sectional	31,435	Male	42.3 ± 8.8	Korean male firefighters	Organizational injustice, high job demands	Cross-sectional/NR	Lower back pain, work-related musculoskeletal disorders (WMSDs)
Kim et al. (2013) [47]	Cross-sectional	31,435	Male	42.3 ± 8.8	Korean male firefighters	Lack of reward, high job demands	Cross-sectional/NR	Work-related musculoskeletal disorders (WMSDs)
Damrongsak et al. (2017) [48]	Cross-sectional	298	Male	40.6 ± 9.1 (cases), 39.1 ± 8.8 (controls)	US career firefighters	Job-related stress (JSS)	Cross-sectional/NR	Current back pain
Soteriades et al. (2019) [49]	Cross-sectional	901	Mixed	41.4 ± 9.6	European firefighters	Work-related stress	Cross-sectional/NR	Musculoskeletal problems
Jang et al. (2016) [50]	Cross-sectional	1,217	Mixed	M: 42.9 ± 8.7, F: 34.2 ± 7.2	Korean firefighters	Lack of reward, interpersonal conflict, organizational system	Cross-sectional/NR	Gastroesophageal reflux disease (GERD)
Jang et al. (2017) [51]	Cross-sectional	1,217	Mixed	M: 42.9 ± 8.7, F: 34.2 ± 7.2	Korean firefighters	Job demand (OR 1.79), interpersonal conflict (OR 2.21), lack of reward (OR 2.39)	Cross-sectional/NR	Irritable Bowel Syndrome (IBS) by Rome III criteria, quality of life (WHOQOL-BREF)
Khoshakhlagh et al. (2024) [52]	Cross-sectional	2,339	Mixed (NR)	NR	Firefighters (Iran)	Job stress (HSE), PTSD	Cross-sectional (2022)/NR	Musculoskeletal symptoms (Nordic Musculoskeletal Questionnaire), burnout (MBI), self-reported depressive symptoms (CES-D)
Dodge et al. (2025) [20]	Cross-sectional	44	NR	NR	Firefighters (US)	Years of experience, live fire-training evolution (LFTE)	Cross-sectional/NR	Salivary stress biomarkers: α-amylase (AA), secretory immunoglobulin-A (SIgA), cortisol (CORT)
**6. Physical Performance (Firefighting-specific)**								
Perroni et al. (2015) [53]	Cross-sectional	229	Male	32 ± 8 (range: < 30 to > 45)	Italian professional firefighters	Age-related changes in strength and power	Cross-sectional/NR	Upper Body Strength (1RM bench press), Lower Limb Power (CMJ), Sprint (20 m running time)
Kim et al. (2019) [54]	Cross-sectional	618	Mixed	41.1 ± 8.0	Korean firefighters (4 cities)	Work-related traumatic events	Cross-sectional/NR	Work limitations (physical, psychosocial, environmental work demands)

*NR* Not Reported, *M* Male, *F* Female, *PTEs* Potentially Traumatic Events, *KOSS* Korean Occupational Stress Scale, *JSS* Job Stress Survey, *PSS* Perceived Stress Scale, *CES-D* Center for Epidemiologic Studies Depression Scale, *PHQ* Patient Health Questionnaire, *SBQ-R* Suicidal Behaviors Questionnaire-Revised, *GAD-7* Generalized Anxiety Disorder-7, *IES-R* Impact of Event Scale-Revised, *MBI* Maslach Burnout Inventory, *OLBI* Oldenburg Burnout Inventory, *LBQ* Link Burnout Questionnaire, *AUDIT* Alcohol Use Disorders Identification Test, *PSQI* Pittsburgh Sleep Quality Index, *HRV* Heart Rate Variability, *WMSDs* Work-Related Musculoskeletal Disorders, *GERD* Gastroesophageal Reflux Disease, *IBS* Irritable Bowel Syndrome, *WHOQOL-BREF* World Health Organization Quality of Life-BREF, *HSE* Health and Safety Executive

### Characteristics of the included studies

The 34 original studies were published between 2006 and 2025, investigating the associations of job stress with various physical and mental health outcomes in firefighter populations. The total number of participants across all studies was 87,242, with individual sample sizes ranging from 15 to 45,698 participants (median = 651). The studies included both male and female firefighters, with 15 studies examining both sexes, 12 studies focusing solely on male firefighters, one study involving only female firefighters, and one study including male, female, and transgender firefighters. Age reporting varied across studies; most studies reported mean ages ranging from approximately 25 to 45 years or provided age category distributions (e.g., 20–29 years, 30–39 years, 40–49 years, 50 + years). Studies were conducted across multiple geographic regions, with the majority focused on firefighters within Asia (n = 15; predominantly South Korea), North America (n = 10; primarily United States), Europe (n = 6; including Portugal, Poland, UK, Italy, and the Netherlands), and South America (n = 1). Most studies employed a cross-sectional design (33/34, 97.1%), with only three longitudinal studies (8.8%).

The measurement tools used to assess job stress varied considerably across studies, with 15 different scales employed. The most common scale was the Korean Occupational Stress Scale (KOSS), appearing in 6 studies primarily from South Korea. Other scales included the Job Stress Survey (JSS), Perceived Stress Scale (PSS), and various study-specific questionnaires. For health outcome assessment, 18 separate validated instruments were used, including the Center for Epidemiologic Studies Depression Scale (CES-D) for depression (8 studies), the Maslach Burnout Inventory (MBI) for burnout (3 studies), the Pittsburgh Sleep Quality Index (PSQI) for sleep quality (3 studies), the Alcohol Use Disorders Identification Test (AUDIT) for alcohol use problems (3 studies), and the Posttraumatic Stress Disorder Checklist (PCL-C) for self-reported PTSD symptoms (2 studies).

### Depression and suicidality

Eleven studies examined the association between job-related psychosocial stress and depression or suicidality in firefighters, and the evidence consistently demonstrated that work-related psychosocial stress was associated with a significantly higher likelihood of depressive symptoms and suicidal ideation. The strongest evidence came from the longitudinal study by An, Chung [22], which found that perceived organizational system stress (encompassing organizational fairness, support, and position appropriateness) was associated with a markedly higher odds of depressive symptoms with an odds ratio of 8.03 (95% CI: 1.73, 37.22). Three cross-sectional studies using the CES-D scale observed a positive association between occupational stress and depressive symptoms, with Saijo, Ueno and Hashimoto [23] identifying high variance in workload and high intergroup conflict as the most important factors influencing this association. Regarding suicidality, Park, Kim [28] conducted a nationwide study of 31,435 Korean firefighters and found that perceived job stress was associated with suicidal ideation, while Carpenter, Carpenter [27] observed that the relationship between psychosocial stress and suicidal ideation was present only among firefighters who reported inadequate social support. Stanley, Boffa [29] further found that distress tolerance buffered the association between occupational stress and suicidality. Among female firefighters, Jahnke, Haddock [26] reported that those experiencing the highest level of perceived discrimination and harassment were more than twice as likely to report depressive symptoms (OR = 2.18; 95% CI: 1.56, 3.05) and had elevated risk for suicidal ideation. More recent studies by Zegel, Lebeaut [30] and Rapisarda, Guay [31] confirmed these associations, reporting that resilience and distress tolerance indirectly influence anxiety and depression symptoms in firefighters. Collectively, these findings indicate that organizational system stress, particularly related to fairness and support, has the strongest association with depression, while inadequate social support serves as a critical moderator for suicidal ideation.

### Non-Depressive Mental Health Problems (PTSD, Anxiety, Burnout)

Eight studies investigated the association between job stress and PTSD symptoms, anxiety, or burnout, revealing consistent positive associations across multiple populations and geographic regions. Regarding PTSD symptoms and anxiety, Mitani, Fujita [32] found that job-related stress and traumatic exposure were positively associated with PTSD symptoms and burnout among Japanese fire service workers, while Saijo, Ueno and Hashimoto [33] similarly observed that job stress measured by KOSS significantly predicted PTSD symptoms in urban Japanese firefighters. Among female firefighters, Jahnke, Haddock [26] reported particularly strong associations, with those experiencing the highest level of discrimination and harassment being more than twice as likely to report PTSD symptoms (OR = 2.21; 95% CI: 1.58, 3.09) and anxiety (OR = 2.56; 95% CI: 1.75, 3.75). Concerning burnout, Ângelo and Chambel [35] conducted one of the few longitudinal studies and observed a reciprocal association between organizational demands and burnout: the perception of increased organizational demands predicted burnout, which in turn was associated with an altered perception of job demands. Smith, DeJoy [36] found that work pressure, work stress, and work-family conflict were positively associated with burnout (emotional exhaustion and depersonalization) in US firefighters. Makara-Studzińska, Golonka and Izydorczyk [37] reported that life stress was consistently correlated with most domains of burnout in Polish male firefighters, with self-efficacy serving as a significant moderator. These findings collectively demonstrate that discrimination and harassment have particularly strong associations with PTSD symptoms and anxiety in female firefighters, while burnout shares a reciprocal association with organizational demands over time.

### Alcohol use disorders

Four studies examined the association between job stress and alcohol use disorders in firefighters, consistently demonstrating that perceived stress was associated with an increased likelihood of problematic alcohol use. The largest study, by Kim, Park and Kim [38], involved 7,151 Korean firefighters and found that perceived job stress significantly predicted an increased likelihood of alcohol abuse as measured by the AUDIT-K. Hosoda, Osaki [39] found that a poor perception of the workplace environment was a key factor influencing alcohol dependence among male Japanese firefighters. Jahnke, Haddock [26] observed that female firefighters with the highest level of perceived discrimination and harassment had a clearly elevated risk of alcohol abuse (OR = 1.54; 95% CI: 1.09, 2.17). Arbona, Pao [40] studied Black and Latino male firefighters and found that levels of alcohol abuse rose with levels of perceived general stress. Across these studies, discrimination and harassment emerged as a particularly important risk factor, associated with a 54% increased risk of alcohol abuse among female firefighters.

### Sleep quality

Three studies investigated the association between psychosocial stressors and sleep quality among firefighters, yielding mixed evidence. Haddock, Poston [41] observed that firefighters working longer shifts (48-h shifts) were significantly more likely to experience excessive daytime sleepiness (EDS) compared with those working 24-h shifts; however, EDS was not influenced by the degree of perceived general life stress. Lim, Baek [42] found that many occupational stress factors were correlated with poor sleep quality in a large sample of Korean firefighters, although work-related psychosocial stress was not a significant independent predictor after adjustment for covariates. By contrast, Yook [43] noted a graded association wherein increasing occupational stress (measured by KOSS) was associated with declining sleep quality as measured by the PSQI. The inconsistency in findings may be attributable to differences in sample characteristics, measurement tools, and adjustment for confounding variables such as shift schedule and years of service.

### Physiological parameters and somatic disorders

Eleven studies examined the association between job stress and physiological parameters or somatic disorders, including cardiovascular function, musculoskeletal disorders, and gastrointestinal disorders. Regarding cardiovascular function, Choi, Schnall and Dobson [44] found that increased job demands and 24-h work shifts were associated with modest but significant increases in systolic blood pressure. For heart rate variability (HRV), Shin, Lee [45] found that concerns about occupational climate and organizational system were associated with undesirable changes in certain HRV parameters, whereas Yook [43] observed no significant associations between any of the eight KOSS factors and standard HRV parameters. Concerning musculoskeletal disorders, Kim, Seo [46] found that the risk of lower back pain in Korean firefighters was associated with organizational injustice (OR = 1.53; 95% CI: 1.04, 2.24) and high job demands (OR = 1.55; 95% CI: 1.35, 1.77). Kim, Kim [47] reported that lack of reward (OR = 2.39; 95% CI: 1.08, 5.26) and high job demands (OR = 1.52; 95% CI: 1.35, 1.70) were associated with work-related musculoskeletal disorders (WMSDs). Damrongsak, Prapanjaroensin and Brown [48] did not find that back pain could be predicted by job-related stress, although age and history of back pain were highly predictive. Soteriades, Psalta [49] reported that European firefighters with the greatest level of work-related stress had an increased risk of musculoskeletal problems (OR = 1.52; 95% CI: 1.02, 2.25). Regarding gastrointestinal disorders, Jang, Ryu [50] found that lack of reward (OR = 2.17; 95% CI: 1.21, 3.88) and interpersonal conflict (OR = 2.07; 95% CI: 1.06, 3.51) were associated with increased risk for gastroesophageal reflux disease (GERD). Jang, Ryu [51] found that lack of reward (OR = 2.17; 95% CI: 1.21, 3.88) and interpersonal conflict (OR = 2.07; 95% CI: 1.06, 3.51) were associated with increased risk for gastroesophageal reflux disease (GERD). Recent evidence from Khoshakhlagh, Sulaie [52] studied 2,339 Iranian firefighters and found that high job stress alone increased the probability of musculoskeletal symptoms by 34%, high PTSD symptoms alone increased probability by 34%, and combined high job stress plus high PTSD symptoms increased probability by 37%, with depression having the highest mediation effect among burnout and depression. Overall, organizational factors such as lack of reward, interpersonal conflict, and high job demands were consistently associated with musculoskeletal and gastrointestinal disorders, with odds ratios ranging from 1.52 to 2.39.

### Physical performance

Three studies examined physical performance outcomes in relation to job-related factors. Kim, Jeong [54] reported that exposure to work-related traumatic events was associated with work limitations, with threat or injury to self-showing the strongest association for environmental work demands (OR = 4.89; 95% CI: 1.66, 14.40). Dodge, Coles [20] reported that less experienced firefighters (under 6 years of service) demonstrated greater physiological stress responses, with cortisol increasing by 209.5% following live fire-training evolution compared to a 178.3% increase in experienced firefighters (6 or more years of service), indicating that tenure/experience is a correlate of physiological stress responses. In addition, Perroni, Guidetti [53] examined age-related changes in Italian firefighters, describing a 12% decrease in upper body strength and a 13% decrease in lower limb power between firefighters aged under 30 years and those over 45 years; however, this study was not directly examining job stress as a predictor of performance and is therefore noted as contextual background rather than evidence of a stress-performance association.

A summary table (Table 2) was created to organize findings by outcome domain, presenting key effect sizes (e.g., odds ratios, correlation coefficients) for rapid comparison across studies (Table 2).

**Table 2:** Summary of Key Associations by Outcome Domain

Outcome Domain	Key Psychosocial Stressor	Key Finding (Effect Size)	Study Design (Number of Studies)
**Depression & Suicidality**	Organizational system stress	OR = 8.03 (95% CI: 1.73, 37.22) – longitudinal study	Longitudinal (1), Cross-sectional (10)
	Discrimination & harassment (females)	OR = 2.18 (95% CI: 1.56, 3.05) for self-reported depressive symptoms	Cross-sectional
	Inadequate social support	Moderates stress-suicidality association	Cross-sectional
**Non-Depressive Mental Health (PTSD Symptoms, Anxiety, Burnout)**	Discrimination & harassment (females)	OR = 2.21 (95% CI: 1.58, 3.09) for self-reported PTSD symptoms; OR = 2.56 (95% CI: 1.75, 3.75) for anxiety	Cross-sectional
	Organizational demands	Reciprocal association with burnout	Longitudinal (1), Cross-sectional (7)
**Alcohol Use Disorders**	Perceived job stress	Significant predictor of alcohol abuse	Cross-sectional (4)
	Discrimination & harassment (females)	OR = 1.54 (95% CI: 1.09, 2.17)	Cross-sectional
**Sleep Quality**	Occupational stress (KOSS)	Graded association with poor sleep quality	Cross-sectional (3) – mixed evidence
**Somatic Disorders**	Lack of reward	OR = 2.17–2.39 for GERD and IBS; OR = 2.39 for WMSDs	Cross-sectional (11)
	Interpersonal conflict	OR = 2.07–2.21 for GERD and IBS	Cross-sectional
	High job demands	OR = 1.52–1.55 for WMSDs and lower back pain	Cross-sectional
**Physical Performance**	Work-related traumatic events	OR = 4.89 (95% CI: 1.66, 14.40) for environmental work limitations	Cross-sectional (2)
	Less experience (tenure)	Greater physiological stress responses (209.5% vs. 178.3% cortisol increase)	Cross-sectional

### Study quality

Quality assessment was conducted using the Newcastle–Ottawa Scale (NOS) adapted for cross-sectional studies for the 33 cross-sectional studies, and the NOS for cohort studies for the three longitudinal studies. The quality assessment revealed that 12 studies (32.4%) were classified as good quality (8–10 stars), 21 studies (61.8%) as satisfactory quality (6–7 stars), and 1 study (2.9%) as poor quality (0–5 stars) following our predefined thresholds (Good: 8–10 stars, Satisfactory: 6–7 stars, Poor: 0–5 stars). Specifically, Dodge, Coles [20] received 5 stars and was classified as "Poor." Strengths of the included studies included large sample sizes (multiple nationwide studies with over 30,000 participants), use of validated measurement tools for both exposure and outcome assessment, and adequate statistical analyses. Limitations included the predominance of cross-sectional designs (91.7%), which limits causal inference; lack of sample size justifications in most studies; inadequate reporting of non-respondent characteristics; and insufficient control for potential confounders such as age, years of service, and shift schedule in some studies. Detailed quality assessment results for each study are presented in Table 3.

**Table 3:** Newcastle–Ottawa Scale (NOS) Assessment

Title	Selection (0–5★)	Comparability (0–2★)	Outcome (0–3★)	Total Score (0–10★)	Quality	
**Depression & Suicidality**						
An et al. (2015) [22]	The effect of organisational system on self-rated depression in a panel of male municipal firefighters	★★★★ (4)	★★ (2)	★★ (2)	8	**Good**
Saijo et al. (2007) [23]	Job stress and depressive symptoms among Japanese fire fighters	★★★ (3)	★ (1)	★★ (2)	6	**Satisfactory**
Saijo et al. (2008) [24]	Twenty-four-hour shift work, depressive symptoms, and job dissatisfaction among Japanese firefighters	★★★ (3)	★ (1)	★★ (2)	6	**Satisfactory**
Payne & Kinman (2019) [25]	Job demands, resources and work-related well-being in UK firefighters	★★★ (3)	★★ (2)	★★★ (3)	8	**Good**
Jahnke et al. (2019) [26]	The prevalence and health impacts of frequent work discrimination and harassment among women firefighters in the US fire service	★★★★ (4)	★★ (2)	★★ (2)	8	**Good**
Carpenter et al. (2015) [27]	Social support, stress, and suicidal ideation in professional firefighters	★★★ (3)	★ (1)	★★ (2)	6	**Satisfactory**
Park et al. (2019) [28]	Prevalence and correlates of suicidal ideation in Korean firefighters: A nationwide study	★★★★★ (5)	★★ (2)	★★ (2)	9	**Good**
Stanley et al. (2018) [29]	Occupational stress and suicidality among firefighters: Examining the buffering role of distress tolerance	★★★ (3)	★★ (2)	★★ (2)	7	**Satisfactory**
Zegel et al. (2025) [30]	Resilience and distress tolerance among firefighters: Associations with anxiety and depression symptoms	★★★ (3)	★★ (2)	★★ (2)	7	**Satisfactory**
Rapisarda et al. (2024) [31]	Longitudinal assessment of psychological distress and its determinants in a sample of firefighters based in Montreal, Canada	★★★★ (4)	★★ (2)	★★★ (3)	9	**Good**
**Non-depressive mental health problems (PTSD, Anxiety, Burnout)**						
Mitani et al. (2006) [32]	Impact of posttraumatic stress disorder and job-related stress on burnout: A study of fire service workers	★★★ (3)	★ (1)	★★ (2)	6	**Satisfactory**
Saijo et al. (2012) [33]	Post-traumatic stress disorder and job stress among firefighters of urban Japan	★★★ (3)	★ (1)	★★ (2)	6	**Satisfactory**
Angelo & Chambel (2015) [35]	The reciprocal relationship between work characteristics and employee burnout and engagement: A longitudinal study of firefighters	★★★★ (4)	★★ (2)	★★★ (3)	9	**Good**
Smith et al. (2019) [34]	Impact of work pressure, work stress and work-family conflict on firefighter burnout	★★★ (3)	★★ (2)	★★ (2)	7	**Satisfactory**
Makara-Studzińska et al. (2019) [37]	Self-efficacy as a moderator between stress and professional burnout in firefighters	★★★ (3)	★ (1)	★★ (2)	6	**Satisfactory**
**Alcohol Use Disorders**						
Kim et al. (2018) [38]	The mediation effect of PTSD, perceived job stress and resilience on the relationship between trauma exposure and the development of depression and alcohol use problems in Korean firefighters	★★★★ (4)	★★ (2)	★★ (2)	8	**Good**
Hosoda et al. (2012) [39]	Evaluation of relationships among occupational stress, alcohol dependence and other factors in male personnel in a Japanese local firefighting organization	★★★ (3)	★ (1)	★★ (2)	6	**Satisfactory**
Arbona et al. (2017) [40]	Perceived stress in Black and Latino male firefighters: Associations with risk and protective factors	★★★ (3)	★ (1)	★★ (2)	6	**Satisfactory**
**Sleep Quality**						
Haddock et al. (2013) [41]	Excessive daytime sleepiness in firefighters in the central United States	★★★ (3)	★★ (2)	★★ (2)	7	**Satisfactory**
Lim et al. (2014) [42]	Factors related to sleep disorders among male firefighters	★★★★ (4)	★ (1)	★★ (2)	7	**Satisfactory**
Yook (2019) [43]	Firefighters' occupational stress and its correlations with cardiorespiratory fitness, arterial stiffness, heart rate variability, and sleep quality	★★★ (3)	★★ (2)	★★★ (3)	8	**Good**
**Physiological Parameters & Somatic Disorders**						
Choi et al. (2016) [44]	Twenty-four-hour work shifts, increased job demands, and elevated blood pressure in professional firefighters	★★★ (3)	★ (1)	★★ (2)	6	**Satisfactory**
Shin et al. (2016) [45]	Factors related to heart rate variability among firefighters	★★★ (3)	★ (1)	★★ (2)	6	**Satisfactory**
Kim et al. (2016) [46]	Nationwide firefighter survey: the prevalence of lower back pain and its related psychological factors among Korean firefighters	★★★★ (4)	★★ (2)	★★ (2)	8	**Good**
Kim et al. (2013) [47]	Relationship between occupational stress and work-related musculoskeletal disorders in Korean male firefighters	★★★★ (4)	★★ (2)	★★ (2)	8	**Good**
Damrongsak et al. (2017) [48]	Predictors of back pain in firefighters	★★★ (3)	★ (1)	★★ (2)	6	**Satisfactory**
Soteriades et al. (2019) [49]	Occupational stress and musculoskeletal symptoms in firefighters	★★★ (3)	★ (1)	★★ (2)	6	**Satisfactory**
Jang et al. (2016) [50]	Psychological factors influence the gastroesophageal reflux disease (GERD) and their effect on quality of life among firefighters in South Korea	★★★ (3)	★★ (2)	★★★ (3)	8	**Good**
Jang et al. (2017) [51]	Psychological factors influence the irritable bowel syndrome and their effect on quality of life among firefighters in South Korea	★★★ (3)	★★ (2)	★★★ (3)	8	**Good**
Khoshakhlagh et al. (2024) [52]	Relationships between job stress, post-traumatic stress and musculoskeletal symptoms in firefighters and the role of job burnout and depression mediators	★★★ (3)	★★ (2)	★★ (2)	7	**Satisfactory**
**Physical Performance**						
Perroni et al. (2015) [53]	Age-related changes in upper body strength and lower limb power of professional Italian firefighters	★★★ (3)	★ (1)	★★ (2)	6	**Satisfactory**
Kim et al. (2019) [54]	The association of the exposure to work-related traumatic events and work limitations among firefighters	★★★ (3)	★ (1)	★★ (2)	6	**Satisfactory**
Dodge et al. (2025) [20]	Impact of years of experience on the stress responses to firefighting	★★ (2)	★ (1)	★★ (2)	5	**Poor**

### Certainty of evidence

This analysis did not include a formal assessment of reporting bias, such as through searching clinical trial registries or assessing publication bias via funnel plots, due to the substantial methodological heterogeneity across studies and the predominance of cross-sectional designs. Additionally, the overall certainty of the evidence for each outcome was not evaluated using established frameworks such as GRADE (Grading of Recommendations Assessment, Development and Evaluation) because the majority of included studies were observational and the evidence base lacked sufficient randomized controlled trials or longitudinal studies to support GRADE assessment. Future systematic reviews with a larger number of longitudinal studies may benefit from GRADE or similar certainty assessment frameworks.

## Discussion

This systematic review synthesizes evidence from 34 original studies examining the associations of job stress with the physical and mental health of firefighters, involving a total of 87,242 participants. The primary outcomes were categorized into six domains: depression and suicidality, non-depressive mental health problems (PTSD symptoms, anxiety, burnout), alcohol use disorders, sleep quality, physiological parameters and somatic disorders, and physical performance. Key findings indicate that work-related psychosocial stressors, particularly organizational system stress (lack of fairness, inadequate support), interpersonal conflict, discrimination and harassment, and high job demands, are consistently associated with adverse health outcomes across multiple domains. The strongest associations were observed for organizational system stress with self-reported depressive symptoms (OR = 8.03 in the longitudinal study), discrimination and harassment with self-reported PTSD symptoms (OR = 2.21) and anxiety (OR = 2.56) in female firefighters, and lack of reward with gastrointestinal disorders (OR = 2.17–2.39) and musculoskeletal disorders (OR = 2.39). Longitudinal evidence from three studies confirmed reciprocal associations between organizational demands and burnout. Overall, this review demonstrates that job stress is associated with pervasive associations across multiple physiological systems, extending well beyond psychological distress.

A particularly robust finding across multiple studies was the central role of organizational system stress in predicting mental health outcomes. The longitudinal study by An, Chung [22] demonstrated that firefighters who perceived their organizational system as unfair or lacking in support had an eight-fold higher odds of depressive symptoms. This finding is consistent with the broader occupational health literature establishing that perceived organizational justice is a powerful predictor of mental health outcomes [6]. The mechanism underlying this association may involve the erosion of psychological resources; when firefighters perceive that their efforts are not fairly rewarded or that organizational decisions are arbitrary, they experience helplessness and reduced self-efficacy, known precursors to depression [7]. Furthermore, inadequate social support moderated the association between stress and suicidal ideation, suggesting that the absence of buffering resources may be as important as the presence of stressors themselves [14]. From a practical standpoint, interventions aimed at improving organizational fairness, enhancing supervisor support, and fostering a positive workplace climate may be more effective in preventing depression than interventions focused solely on individual coping skills [8].

The findings related to discrimination and harassment among female firefighters reveal significant gender-specific vulnerabilities within the fire service. Jahnke, Haddock [26] demonstrated that female firefighters experiencing the highest levels of discrimination and harassment had more than double the odds of depressive symptoms, PTSD symptoms, and anxiety, and 54% increased odds of alcohol abuse. These findings are particularly concerning given that women remain a minority in the fire service and may face unique occupational challenges, including social isolation and persistent gender-based stereotypes [42]. The magnitude of these associations (ORs ranging from 1.54 to 2.56) suggests that discrimination and harassment are significant occupational hazards with measurable health consequences. From a policy perspective, these findings underscore the need for zero-tolerance policies regarding workplace harassment, confidential reporting mechanisms, and gender-sensitive mental health support services.

The longitudinal study by Ângelo and Chambel [35] provided unique insights into the reciprocal association between organizational demands and burnout, demonstrating a vicious cycle wherein organizational stressors are associated with burnout, and burned-out firefighters subsequently perceive their work environment as even more demanding. This finding aligns with conservation of resources theory, which posits that stress occurs when resources are threatened or lost. Makara-Studzińska, Golonka and Izydorczyk [37] identified self-efficacy as a significant moderator of the stress-burnout association, suggesting that interventions aimed at enhancing firefighters' belief in their ability to cope with job demands may be protective against burnout.

Regarding somatic disorders, eleven studies consistently demonstrated that organizational factors such as lack of reward, interpersonal conflict, and high job demands are associated with musculoskeletal and gastrointestinal disorders. The finding that lack of reward and interpersonal conflict where the strongest predictors of irritable bowel syndrome suggests that psychosocial factors can influence gastrointestinal function through the brain-gut axis. Similarly, the association between high job demands and lower back pain may be explained by stress-induced muscle tension and reduced recovery time between shifts. The recent Bayesian network analysis by Khoshakhlagh, Sulaie [52] demonstrated that combined high job stress and high PTSD symptoms increased the probability of musculoskeletal symptoms, indicating that psychological and physical health outcomes are interconnected and may exert synergistic effects.

Firefighters are exposed to a unique constellation of occupational stressors that distinguish them from other first responders and general occupational populations. The para-military organizational structure, 24-h shift schedules, culture of stoicism, and increasing workforce diversification create occupation-specific vulnerabilities that shape how job stress manifests and impacts health. For example, chronic sleep disruption due to shift work may compound the physiological effects of organizational stress, while the suppression of emotional expression may delay help-seeking and exacerbate psychological distress. The somatic disorders observed in this review particularly musculoskeletal and gastrointestinal complaints reflect the physical demands of firefighting, including heavy protective gear, prolonged standing, and irregular meal patterns, which may interact with psychosocial stressors to produce cumulative strain. Furthermore, the underrepresentation of women and racial/ethnic minorities in the fire service, combined with experiences of discrimination and harassment, adds a layer of social identity-based stress that is not present for the majority of male firefighters. These occupation-specific factors underscore the need for tailored interventions that address not only individual coping but also the organizational culture and structural conditions that shape firefighter health.

Several protective factors emerged as consistent moderators of the stress-health association. Social support buffered against depression, suicidal ideation, and burnout. Self-efficacy moderated the stress-burnout association, while distress tolerance buffered the association between occupational stress and suicidality. These findings suggest that resilience training programs focusing on self-esteem, social support, distress tolerance, and positive workplace perceptions may be beneficial for firefighters.

### Limitations and future directions

Several limitations must be acknowledged. First, the predominance of cross-sectional designs (91.7%) severely limits causal inference. Second, substantial methodological heterogeneity across studies (15 different stress scales, 18 different outcome tools) precluded meta-analysis. Third, the predominance of studies from South Korea and the United States limits the generalizability of the findings to other cultural and organizational contexts. Fourth, the underrepresentation of female firefighters limits the generalizability of gender-specific conclusions. Fifth, the lack of objective stress measures in most studies means that physiological mechanisms remain speculative. Sixth, the certainty of evidence is limited because formal GRADE assessment was not feasible due to the predominance of cross-sectional designs, absence of randomized controlled trials, and substantial methodological heterogeneity. Seventh, the review's search was limited to English-language publications, potentially introducing language and publication bias. These limitations collectively suggest that the findings are most applicable to male-dominated firefighter populations in Western and East Asian contexts, and may not fully represent the experiences of female firefighters, minority groups, or firefighters in low- and middle-income countries.

Future research should prioritize: (1) large-scale prospective longitudinal studies with adequate follow-up to establish temporal relationships and causality, given the predominance of cross-sectional designs; (2) standardized measurement tools for both stress and outcomes to facilitate cross-study comparisons and enable meta-analytic synthesis; (3) objective stress measures (cortisol, inflammatory biomarkers, heart rate variability) to elucidate biological embedding, particularly given the lack of such measures in the current evidence; (4) oversampling of female firefighters and sex-stratified analyses, as the current evidence on female firefighters is limited and suggests heightened vulnerability; (5) randomized controlled trials of organizational-level interventions (supervisor support training, fairness-enhancing policies) and individual-level interventions (resilience training, mindfulness-based stress reduction), building on the protective factors identified in this review; (6) examination of synergistic effects of multiple stressors; (7) development of a standardized, cross-culturally applicable firefighter-specific job stress measure; and (8) adherence to STROBE guidelines to improve reporting quality, as many included studies had inadequate reporting of non-respondents and sample size justifications.

## Conclusions

This systematic review indicates that job stress is associated with clinically significant adverse health outcomes in firefighters. The strongest associations include organizational stress linked to depression, discrimination/harassment linked to PTSD symptoms and anxiety (especially in females), and lack of reward linked to somatic disorders. Protective factors such as social support, self-efficacy, and resilience appear to buffer these associations. However, the available evidence is predominantly cross-sectional, which limits causal inference; therefore, the findings suggest associations rather than causal effects. Despite this limitation, consistent findings across geographic regions and supportive longitudinal evidence support the presence of clinically meaningful associations between job stress and health outcomes in this population. Fire service administrators should implement organizational interventions (fairness, conflict reduction, anti-discrimination policies) alongside individual resilience training. Protecting firefighter health through evidence-based stress prevention is an occupational priority and a public health imperative.

## Supplementary Information

Supplementary Material 1.

Supplementary Material 2.

## Data Availability

The datasets used and/or analyzed during the current study are available from the corresponding author on reasonable request.
